# Technologies for deodorization of malodorous gases

**DOI:** 10.1007/s11356-019-04195-1

**Published:** 2019-02-04

**Authors:** Izabela Wysocka, Jacek Gębicki, Jacek Namieśnik

**Affiliations:** 1 Faculty of Environmental Sciences, Department of Environmental Engineering, University of Warmia and Mazury in Olsztyn, 117 Warszawska St., 10-701 Olsztyn, Poland; 2 Faculty of Chemistry, Department of Process Engineering and Chemical Technology, Gdańsk University of Technology, 11/12 G. Narutowicza Str., 80-233 Gdańsk, Poland; 3 Faculty of Chemistry, Department of Analytical Chemistry, Gdańsk University of Technology, 11/12 G. Narutowicza Str., 80-233 Gdańsk, Poland

**Keywords:** Odor, Malodorous substances removal, Malodorous gases emission limitation, Deodorization

## Abstract

There is an increasing number of citizens’ complaints about odor nuisance due to production or service activity. High social awareness imposes pressure on entrepreneurs and service providers forcing them to undertake effective steps aimed at minimization of the effects of their activity, also with respect to emission of malodorous substances. The article presents information about various technologies used for gas deodorization. Known solutions can be included into two groups: technologies offering prevention of emissions, and methodological solutions that enable removal of malodorous substances from the stream of emitted gases. It is obvious that the selection of deodorization technologies is conditioned by many factors, and it should be preceded by an in-depth analysis of possibilities and limitations offered by various solutions. The aim of the article is presentation of the available gas deodorization technologies as to facilitate the potential investors with selection of the method of malodorous gases emission limitation, suitable for particular conditions.

## Introduction

Malodorous substances, similarly to other types of pollutants emitted to the environment, can be of natural and anthropogenic origin. In majority of cases, the environment copes with the pollution of natural origin; however, different aspects of human activity cause an increase in anthropopressure. This is exemplified by emissions of gaseous components with toxic properties, which can additionally be characterized by an unpleasant odor. Such components are called malodorous substances. Their sources can include:

- Industrial production (e.g., production of phosphoric acid, nitrogen fertilizers, paper, etc.) (Boumnijel et al. 2016)
- Sewage treatment plants (e.g., emissions from the grid and sieve hall, drain plots, lagoons, sludge treatment rooms, etc.) (Lewkowska et al. 2016; Zhou et al. 2016)
- Municipal landfill sites (Lucernoni et al. 2016)
- Livestock and poultry production (e.g., henhouses, barns, etc.) (Van der Heyden et al. 2015)
- Food processing plants (e.g., coffee roasting plants, sugar refineries, slaughter and meat packing plants and rendering facilities, etc.) (Lee et al. 2013; Qamaruz-Zaman et al. 2015; Qamaruz-Zaman and Milke 2012)
- Gastronomy (Lee et al. 2013; Ni et al. 2015)
- Waste treatment process, e.g., compost process (Wang et al. 2015).

Emission of malodorous gases, apart from undoubtedly unfavorable influence on health and life of living organisms, causes discomfort and is the reason for complaints from inhabitants who live near emission sources (Yun and Seo 2013). Exposure to odors can cause, e.g., tension, depression, anger, vigor, fatigue, confusion, total mood disturbance (Schiffman et al. 1994). Thus, it becomes necessary to:

- Identify sources of malodorous substances emissions
- Assessment of odor sources (e.g., concentration, emission rates, intensity)
- Elimination of sources of malodorous substances or their removal from the exhaust gas stream
- Assessment of effectiveness of applied technology

**Fig. 1:**
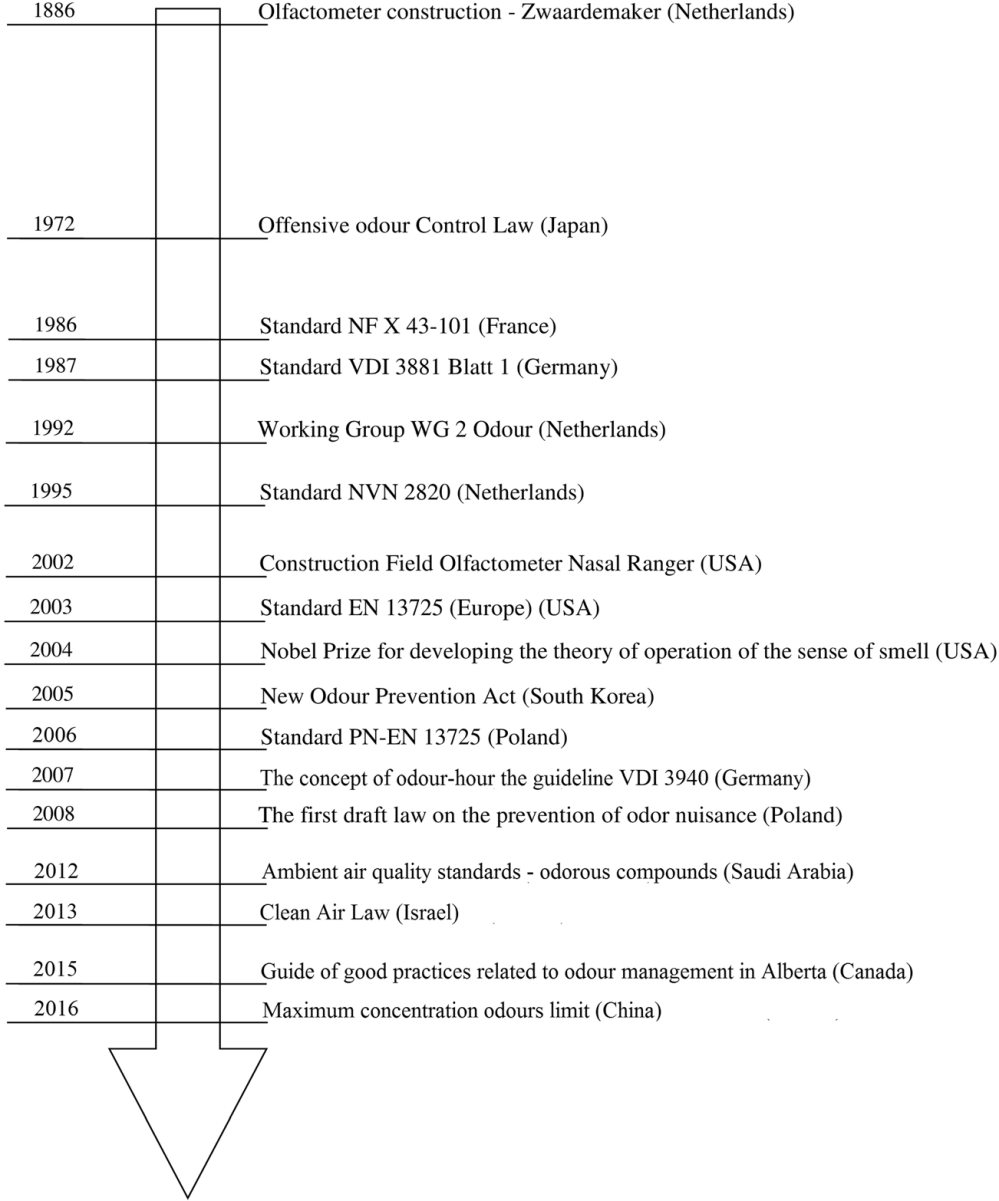
Chosen milestones in development of the legal acts concerning the problem of odor nuisance

Although there are still many techniques available and there exist disputes between scientists concerning methodologies of measurements execution and prediction of odor nuisance in the environment (Brancher et al. 2017; Laor et al. 2014), the treatment of malodorous gases becomes nearly a necessity, facing progressing urbanization, intensification (increase in the efficiency) of animal breeding processes, and constantly developing industrial and services activity. The first attempts of creation of the legal acts concerning the problem of odor nuisance date back to the 60–90s of the twentieth century (Bokowa 2010; Brancher et al. 2017; Guillot et al. 2012; Sowka 2010). Figure 1 presents some the milestones in development of the legal acts pertaining to the problem of odor nuisance in the world.

**Fig. 2:**
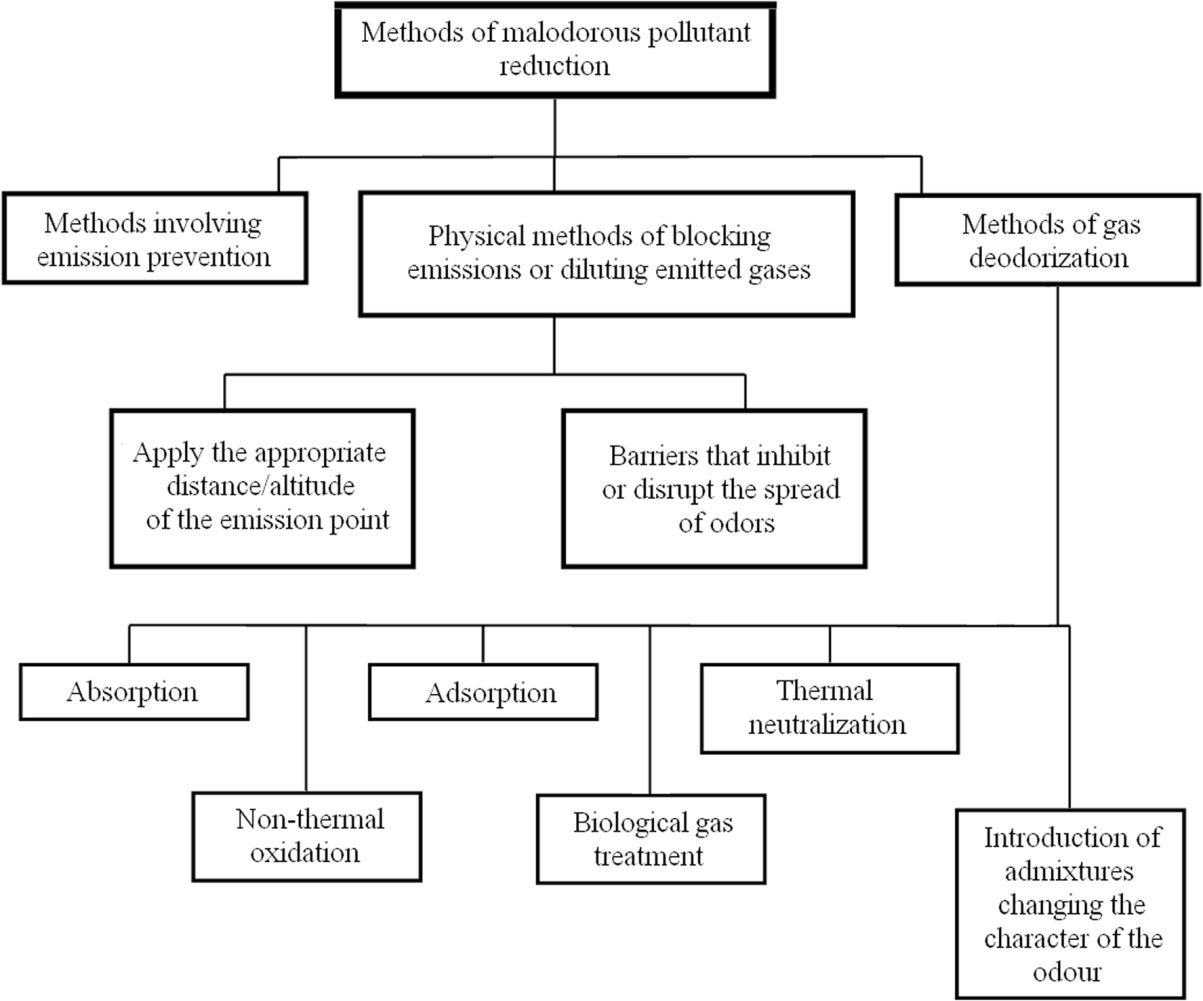
Common methods of limiting pollution with malodorous substances

Up till now, Poland has not passed the law on odor nuisance prevention. Despite legislation problems, there is a number of technologies enabling lowering emission of malodorous substances to the environment. Known technologies of limiting pollution with malodorous substances can be generally included into two main groups: methodological solutions ensuring possibility of preventing malodorous substances emissions (Fig. 2), i.e., preventive technologies and technologies involving deodorization of exhaust gases (Jianming et al. 2014; Wierzbińka and Modzelewski 2015; Rybarczyk et al. 2019).

Preventive methodologies are aimed at preventing or mitigating the occurrence of odor-generating pollution. For example, they are used, e.g.:

1. For animal keeping and breeding by:

- the use of appropriate feed (Gutarowska et al. 2014; Jugowar and Piotrkowski 2012; Matusiak et al. 2016; Maurer et al. 2016)
- the use of urine/feces segregation systems (Maurer et al. 2016)
- the use of appropriate breeding systems, e.g., battery cages, littered ground (Gutarowska et al. 2014; Jugowar and Piotrkowski 2012)

2. In wastewater treatment processes by:

- the use of iron compounds, which promote the formation of free hydroxyl radicals, they increase the redox potential and cause precipitation of sulfur compounds dissolved in wastewater (Jefferson et al. 2002; Jugowar and Piotrkowski 2012)
- the use of chemical compounds, which increase the redox potential in wastewater (above 50 mV) by, e.g., aeration or the use of oxidizing agents such as ozone, oxygen, chlorine, hydrogen superoxide, potassium permanganate, and nitrites (Jugowar and Piotrkowski 2012; Ksibi 2006),
- the use of bactericides reducing the activity of bacteria, which contribute to the increase in intensity of the formation of odor-generating substances (Jugowar and Piotrkowski 2012)
- the use of growth stimulators of microorganisms, which contribute to the inhibition of processes of releasing waste from wastewater (Jugowar and Piotrkowski 2012).

3. In solid waste storage processes:

- shortening of raw waste storage period (Wang et al. 2015)
- avoiding exposure to high temperature (especially in the summer) (Wang et al. 2015)

Prevention of formation and emissions of malodorous substances is sometimes difficult or even impossible to accomplish. The problem of odor nuisance can be limited or eliminated also via application of physical methods consisting in emission blocking or dilution of emitted gases, for instance. This group engulfs all kinds of natural barriers (planting of trees and bushes), artificial barriers (covering of odor emitting areas, for example aeration tanks in sewage treatment plants or solid waste landfills), as well as localization of odor emitting objects within proper distance from residential areas or release of malodorous gases through stacks of sufficient height (Maurer et al. 2016). Also these types of action are not always satisfactory; therefore, it becomes necessary to use an appropriate deodorization technology of gases, which have already been formed. The article presents literature information about known solutions, already implemented in technological practice as well as about proposals of new technologies, which are often still the subject of research. Obviously, the best way to prevent emission of malodorous gas pollutants is to eliminate them at the source. It is not always possible and sometimes not economically reasonable; then deodorization of the emitted gases is an alternative. Malodorous substances, apart from hydrogen sulphide and ammonia, are most frequently organic compounds, so their deodorization involves the techniques utilized for elimination or neutralization of this group of compounds. The techniques presented in Fig. 2 are shortly characterized below.

Gas deodorization by means of the absorption method uses solubility of odor-generating pollutants in the absorption liquid. To increase the intensity of the absorption process of specific odor-forming pollutions, an appropriate absorbent is selected, among other things (Freudenthal et al. 2005). However, the problem of absorbent regeneration or disposal remains. In the technologies based on the use of adsorption phenomena, solid sorbents are employed—their surface adsorbs pollutants. A serious disadvantage of this technology involves, among other things, the necessity to regenerate the deposit with a large quantity of gases (hot air or water steam), which causes subsequent dilution of pollutants, which have already been “collected” (Cartellieri et al. 2005). Thermal neutralization processes mostly include odorant incineration in the stream of air or oxygen. During the process of thermal neutralization of odor-generating compounds, the structure of the compound is destroyed, which eliminates its aromatic properties. Incineration ensures a relatively high percentage of removal of all kinds of organic compounds, to which the majority of odorants belong. However, this is a relatively expensive process (Schlegelmilch et al. 2005b), especially when it is necessary to dispense the inflammable gas to ensure an appropriate combustion temperature or if an appropriate catalyst needs to be added. Non-thermal oxidation processes have also found application in the elimination of malodorous compounds. This group includes process, in which the following are used: (Mielcarek et al. 2009; Yao and Feilberg 2015; Yet-Pole 2004; Zhu et al. 2017):

- Oxidizing compounds
- Oxidizing compounds using catalysts
- UV radiation
- Plasma
- Combination of the aforementioned methodological solutions

Oxidation processes are often combined with absorption processes. In this case, gases are treated due to absorption and the oxidation process occur in the absorption liquid. Biological treatment of malodorous gases can be used if components of this gas are biodegradable. This technology makes it possible to destroy the structure of the malodorous compound. Just like the incineration process, it does not cause substance transfer to another medium. The costs of biological treatment processes are very often much lower than those of alternative processes of treatment of malodorous gases (Ergas and Cárdenas-González 2004).

In some cases, (when odorants occur in low concentrations and are not toxic), the methods of odor masking or neutralization are used, which involve introduction of admixtures into the gas stream (or possibly into the room, in which odors occur) (Mielcarek et al. 2009; Piecuch et al. 2011). Nature of malodorous substances emission results in the fact that not all deodorization techniques exhibit equal usefulness. The next chapters describe principles of operation of the devices employed in these techniques, their advantages and disadvantages. Attention is also paid to economic factor and effectiveness of malodorous substances removal.

Determination of effectiveness of applied deodorization requires thorough gas analysis at inlet and outlet of an installation. It is not always sufficient. Level of odor nuisance perceived by people depends not only on odorants concentration but also on their hedonic quality. An odor can be a desirable phenomenon when it is pleasant, appears with moderate frequency and has moderate intensity. Odorimetric procedures yield many controversies, especially in case of emission measurements. Many countries differ in attitude to this problem, from trivializing and ignoring to implementation of detailed legal regulations (Brancher et al. 2017). Nevertheless, there is still a necessity to undertake the actions aimed at limitation of malodorous gases emission.

## Deodorization of malodorous gases

### Absorption of malodorous substances

Absorption is a process of absorbing a gaseous substance (absorbate) by a liquid or solid (absorbent), i.e., exchange of mass between the gaseous and liquid or solid phase. The aim of absorption is separation of components of the gaseous mixture by removing one or several ingredients from it. Absorption does not require high investment and operation means. It is also applied to deodorization of malodorous gases. Most frequently, it constitutes one of the stages of the entire process of flue gases purification. Its significant advantage is a possibility of malodorous gases removal without the need of preliminary dust extraction process (Szynkowska et al. 2009). This problem occurs particularly in case of purification of the ventilation gases from animal farms. These gases are characterized by high content of dust and aerosols. According to Cai et al. (2006), significant odor nuisance associated with these gases is directly connected with the presence of dust and aerosols, which play a key role in propagation of malodorous pollutants. Absorption, as opposed to adsorption, is a process taking place in the entire volume of the absorbent (Schlegelmilch et al. 2005b). The transport of the absorbate mass mostly depends on the contact surface between the gas being treated and absorbent and on solubility of the absorbate in the absorbent. The process of dissolving in equilibrium is described by the Henry’s law (Schlegelmilch et al. 2005b). According to this law, at a constant temperature, the dependence of the partial pressure (vapor pressure) of the gaseous ingredient over the solution is directly proportional to its concentration in the solution (Eq. 1).

1 $$ {p}_A=H\bullet {x}_A $$

where:

p_A_: Partial pressure (vapor pressure) of ingredient “*A*” in the gas in equilibrium [Pa]
*H*: Henry’s constant [Pa]
*x*_*A*_: Concentration (mole fraction) of ingredient “*A*” in the gas in equilibrium

The value of Henry’s constant depends on the process temperature, on the type of the absorbed ingredient, and the type of solvent. Table 1 presents the values of Henry’s constant for hydrogen sulphide, ammonia, and the main representatives of malodorous volatile organic compounds in case of absorption in water. Moreover, the table contains the values of olfactory threshold for these compounds expressed in ppm *v*/*v* as well as the character of odor sensing (Gebicki et al. 2016; Lu et al. 2015).

**Table 1:** Values of Henry’s constant and olfactory threshold for selected malodorous compounds (Amoore and Hautala 1983; Sander 2015)

Malodorous compounds	Henry constant (atm)	Threshold level of odor identification (ppm)	Type of odor
Hydrogen sulphide	550	0.00041	Rotten eggs
Methanethiol	140	0.00007	Rotten cabbage, garlic
Dimethyl sulphide	110	0.003	Rotten vegetables, garlic
Carbon disulphide	1000	0.21	Rotten vegetables
Ammonia	0.95	17	Sharp, pungent
Methylamine	0.6	4.7	Fish
Dimethylamine	1.3	0.34	Fish
Acetone	1.8	42	Fruity, sweet
Acetaldehyde	3.7	0.0015	Fruity, apple
Formaldehyde	0.018	0.8	Pungent, stifling
Acetic acid	0.01	0.48	Vinegar
Butanoic acid	0.03	0,004	Rancid, odor of sweat
Acrylonitrile	6.1	1.6	Ether smell

Absorption process also requires analysis of kinetics of transport between gas phase and absorbent in order to determine contact time, minimize device dimensions, or process cost.

Magnitude of mass transfer flux can be determined based on the following equation (Eq. 2):

2 $$ \dot{n}={K}_g\bullet A\bullet \Delta  \pi $$

where:

$$ \dot{n} $$
Magnitude of mass transfer flux
*K*_*g*_: Mass transfer coefficient (dependent on mass transfer coefficients in gas and liquid phases as well as on gas-liquid equilibrium constant)
*A*: Surface area of mass transfer
*∆π*: Mean driving force.

Mass transfer surface area depends on type and dimensions of applied absorber. Driving force of the process is dependent on a difference of ingredient concentration in gas under purification and in the equilibrium state (according to the Henry’s law defined for actual concentration of an ingredient in absorption liquid). Mass transfer coefficient is influenced by many factors, including diffusion coefficients, viscosity, density, and flow character of both gas under purification and absorption liquid.

The selection of an appropriate absorbent, absorber design, and process parameters have a considerable influence on the effectiveness of the absorption process (Freudenthal et al. 2005). The absorption process can be carried out via simple dissolution of the pollutants in water (physical absorption). However, the physical absorption is not highly efficient process. The efficiency usually does not exceed 85% (Szynkowska et al. 2009). The problem connected with the absorption processes is onerous waste. After the absorption process, the absorption liquid should undergo the regeneration process or be replaced (Buonicore 1992; Schlegelmilch et al. 2005b). One of the regeneration methods is the use of microorganisms (e.g., bioscrubbers). The absorption liquid is often “enriched” with chemical compounds, which react with absorbed gas (chemical absorption). In this case, the regeneration is conducted by removing reaction products from the absorbent and topping up the chemicals used (Schlegelmilch et al. 2005b). The regeneration can be also performed in-situ, for instance using an electrochemical cell (Govindan and Moon 2013). Comparing both approaches, the chemical absorption is usually more effective than the physical absorption. It causes degradation of the adsorbed compounds, thus increasing a driving force of mass transfer (Boumnijel et al. 2016).

**Fig. 3:**
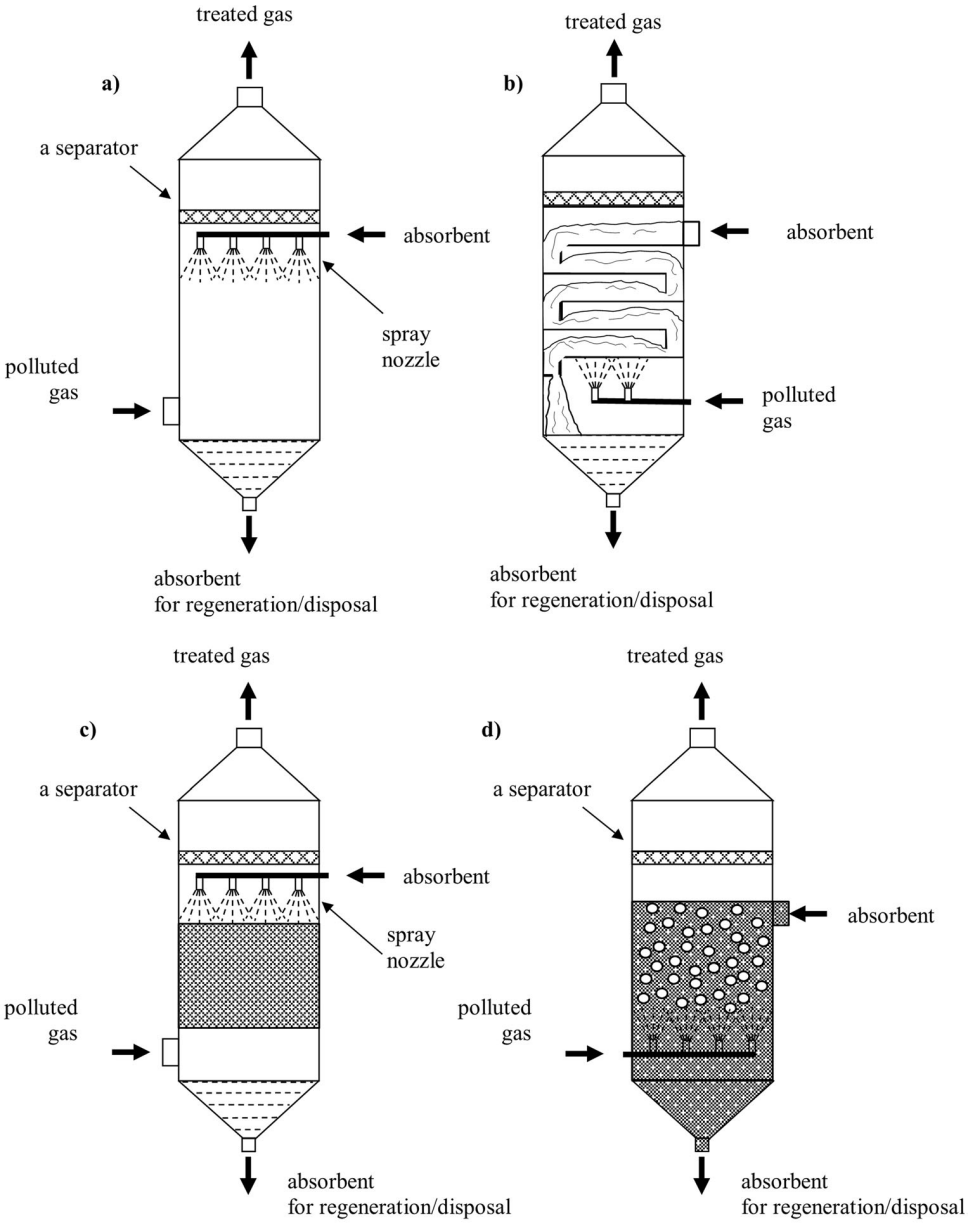
Typical absorber designs: **a** spray scrubber, **b** plate column, **c** packed column, and **d** barbotage column

During deodorization processes, the following substances can be used, for example ozone (O_3_), chlorine (Cl_2_), hydrogen superoxide (H_2_O_2_), sodium hypochlorite (NaOCl), diluted sodium hydroxide, diluted potassium hydroxide, sulfuric acid, or chlorinated seawater (Hahne and Vorlop 2001; Schlegelmilch et al. 2005b). The chemical absorption is frequently used for removal of the pollutants containing sulfur compounds (Biard et al. 2010; Boumnijel et al. 2016; Vega et al. 2014; Vilmain et al. 2014). The most popular is the Claus method and its modifications. It is often used when there is high concentration of hydrogen sulphide (H_2_S) in the gas (Busca and Pistarino 2003; Mokhatab and Poe 2012). Efficiency of this process is at the level of 90–95% for the systems with two-stage catalytic conversion and at the level of 95–98% for the systems with three-stage catalytic conversion (Mokhatab and Poe 2012). The investigations carried out on a semi-technical scale using concurrent method Aquilair Plus™ with aqueous solution of sodium hypochlorite and soda lye revealed 95% efficiency of hydrogen sulphide (H_2_S) removal from the gases originating from a sewage treatment plant (Biard et al. 2010). The flow velocity was 12 m/s and the time of contact was 30 ms. A group of gases containing sulfur compounds also engulfs the flue gases formed during production of phosphoric acid using the wet gypsum method. Efficiency of the processes is also relatively high. Boumnijel et al. (2016) conducted a pioneering research employing chlorinated seawater in the chemical absorption processes during deodorization of the gases originating from production of phosphoric acid in Tunisia. In this case, the main malodorous pollutants of the flue gases were hydrogen sulphide, mercaptans, and hydrogen fluoride. The highest efficiency 98% was obtained for the volumetric flow rate of gas stream of ca. 15 L/ min, at pH = 11 and for chlorine content 1 g Cl_2_/L. The absorber operated for 2.1 h purifying 1905 L of gas and consuming about 6.3 L of absorbent (Boumnijel et al. 2016). However, efficiency at the level of 98% does not guarantee elimination of the problem of odors (Busca and Pistarino 2003). Due to very low olfactory thresholds of many odorants, even a few ppb concentration levels are insufficient. Thus, application of the absorption processes calls for additional gas purification, for instance during hydrogen sulphide (H_2_S) removal where unpleasant odor becomes sensed already at the concentration of 0.5 ppb (Busca and Pistarino 2003). A serious problem associated with the discussed methods, especially if the process is facilitated with chemical reaction, is corrosion of the elements of installations (Szynkowska et al. 2009). That is why proper selection of materials and design of devices are of upmost importance. The absorption process is conducted in devices called absorbers, the structure of which can be very varied. Spray scrubbers, packed columns, plate columns, and barbotage columns are used (Kośmider et al. 2002; Schlegelmilch et al. 2005b). Thus, for example, spray scrubbers are used when main mass transfer resistance occurs due to the gas. The structure of typical design solutions is presented in Fig. 3. Packed columns (Fig. 3) are usually used when mass transfer resistance is similar on the sides of both phases. On the other hand, if main mass transfer resistance is on the side of the liquid, barbotage columns are used when the absorption liquid is the compact phase and the treated gas is the dispersed phase (Fig. 3).

### Adsorption of malodorous substances

Adsorption is a process, in which malodorous substances will be retained on the surface of the sorbent (a solid). It is superficial sorption, i.e., a process consisting in binding of liquid molecules (of gas or liquid–adsorbent) on the surface by solid molecules (adsorbent). One can distinguish two basic types of adsorption, which are applied in the technologies of deodorization of gas stream containing malodorous ingredients:

- Chemical—(chemisorption active adsorption), the adsorbent is bound with the adsorbent using intermolecular forces of the chemical type
- Physical—the adsorbent is bound with the adsorbent using intermolecular forces of the physical type (van der Waals).
- The most important factors that include the course and effectiveness of the adsorption process include:
- Adsorption capacity—depends on the type, porous structure, and the size of adsorbent and properties of the sorbed substance (partial pressure, process temperature)
- The rate of adsorption equilibrium establishment
- Proper specific surface of the adsorbent—it should be very large, even up to 1000 m^2^/g

**Fig. 4:**
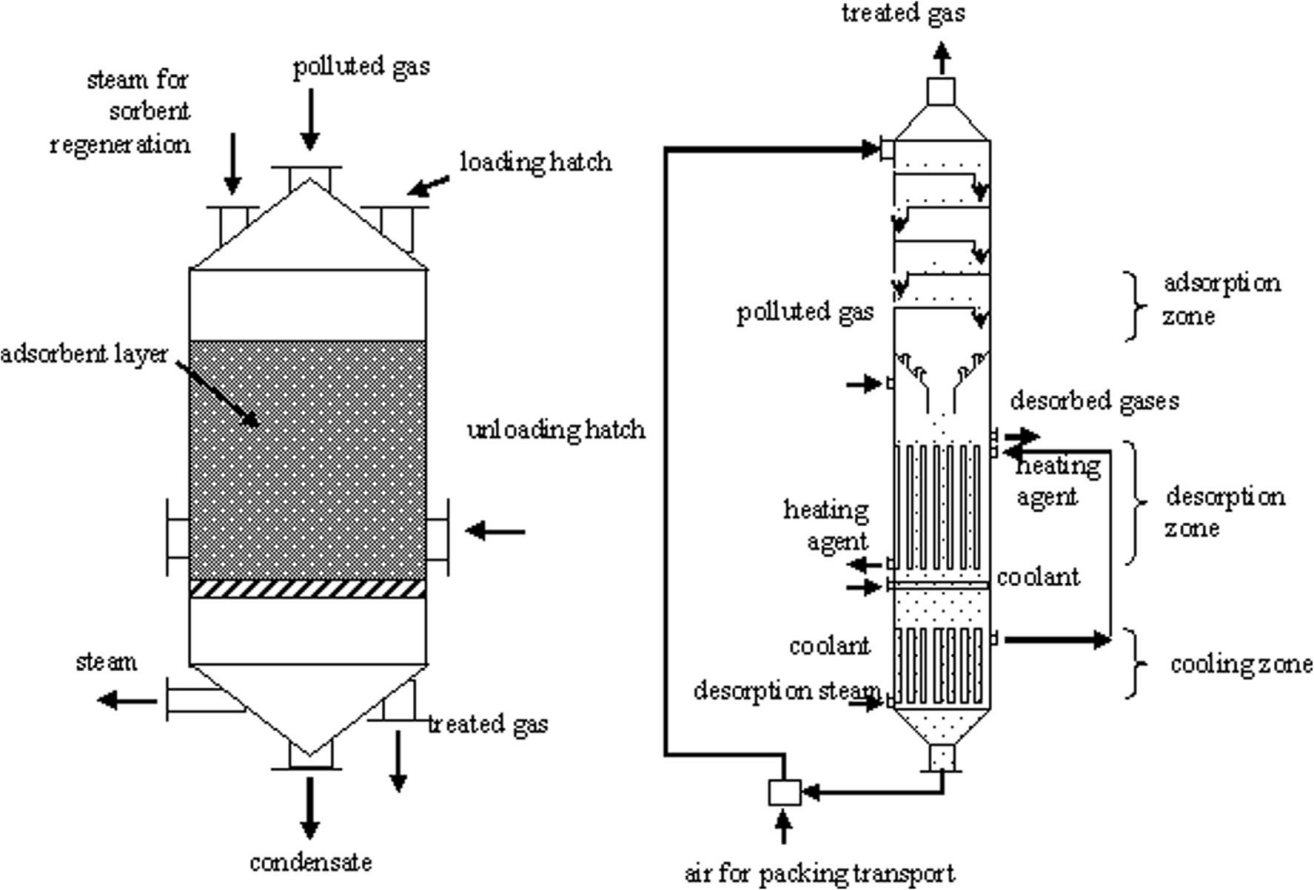
Sample adsorber design: **a** vertical adsorber with a fixed packing layer; **b** plate column with a mobile adsorbent layer

Active carbon, aluminum oxides, silica gels, and zeolites (molecular sieves) usually find application as adsorbents (Schlegelmilch et al. 2005b). All of these (apart from zeolites) have a well-developed uneven surface. Only zeolites are crystalline, regular structures (Buonicore 1992). The adsorbents containing highly developed surface are sensitive to solid pollutants present in the gas under purification. They possess numerous pores in their structure, which are easily blocked by the solid pollutants, thus decreasing contact surface area. Some problems can be also connected with the gas substances binding with sorbent in a permanent way (so-called poisons), which are difficult to remove and block active centers of the adsorbent (Szynkowska et al. 2009). In recent years, the use of specific absorbents selected for a given group of pollutants is recommended (König and Werner 2005). Impregnation of the sorbent (using different types of chemical compounds) is sometimes carried out in order to improve its selectivity or adsorption properties. An example can be impregnation of charcoal with orthophosphoric acid, which improves effectiveness of ammonia and trimethylamine removal (Oya and Iu 2002; Szynkowska et al. 2009). Known design solutions are highly varied, from adsorbers operating periodically in a sorption-desorption system to adsorbers for continuous operation (Fig. 4). Once the adsorption process is finished, it is necessary to employ the used adsorbent or regenerate it. The regeneration process includes desorption, which requires the provision of significant quantities of energy and ensuring appropriate conditions.

Desorption can be conducted using hot air, nitrogen, or water vapor. However, this result in subsequent dilution of pollutants as it requires the use considerable quantities of these gases. Therefore, the final effect is transfer of malodorous pollutants from one stream of gases to another (Cartellieri et al. 2005). The adsorbent can be also regenerated using suitable solvents. Then the pollutants are transferred to liquid phase (Busca and Pistarino 2003). The other method is vacuum desorption. This is, however, connected with generation of a low-pressure system, which, in turn, requires the use of very tight equipment. Desorption under decreased pressure is a bit less troublesome (Busca and Pistarino 2003). Another solution is the use of electrothermal regeneration. It involves heating of an adsorbent deposit with electric current with a low flow rate of an inert gas stream, which rinses pollutants that are being desorbed (Petkovska et al. 2005; Snyder and Leesch 2001; Subrenat et al. 2001). This makes it possible to obtain much smaller gas streams after the regeneration process. Electromagnetic induction also found application in desorption process where temperature of the adsorbent is increased using magnetic field (Bathen and Schmidt-Traub 1999). Microwave radiation is also used as a factor supporting the desorption process (Schlegelmilch et al. 2005b). Both electrothermal, inductive, and microwave-assisted desorption can be used only if adsorbents sensitive to its action are used. Desorption process can be also utilized for recovery of adsorbed ingredients, especially precious ones. In this case, adsorption is treated as a process of concentration of the ingredient diluted in the gas under purification (Szynkowska et al. 2009). In gas deodorization process, adsorption is usually only one of gas treatment stages, in spite of the fact that with application of suitable adsorbent and appropriate process control, it is very efficient method (allows removal of odor-generating pollutants down to the ppb level) (Szynkowska et al. 2009). However, high cost of the adsorbents, limited operation time (due to deactivation by solid particles and poisons), as well as big dimensions of the devices make designers combine adsorption with the other processes (Szynkowska et al. 2009). Adsorption is often combined with the other processes, for example installations together with incineration processes or biological gas deodorization processes (Schlegelmilch et al. 2005b).

### Thermal neutralization of malodorous processes (combustion)

Combustion is a physicochemical process, which is based on a quick reaction of chemical oxidation, which involves rapid combination of the incinerated substance with the oxidizing agent. Oxidation of organic compounds may take place at ambient temperatures; however, this process occurs very slowly and on another route (according to another mechanism). To make incineration process possible, the system should reach an appropriate energy level, i.e., exceed the activation energy.

Three basic techniques (methodological solutions) for this process are known:

- Direct combustion in a flame (temperature approx. 1500 K). It is used for the gases with a very high content of inflammable pollutants. This process is initiated and then it runs spontaneously. Control of the direct combustion process to ensure that only the desired reactions occur is quite difficult. During the direct combustion process, side products, such as nitrogen oxides, carbon oxide, dioxins, multi-ring aromatic hydrocarbons, or soot, are generated.
- Catalytic combustion (400–800 (900) K). The temperatures used in catalytic oxidation are much lower than in thermal combustion. The use of a catalyst lowers the activation energy, i.e., combustion can take place at lower temperatures. This has found application for low concentrations of hydrocarbons in combusted gases. In the catalytic combustion process, the stream of gas contacts the catalyst at an increased temperature. Catalysts are usually applied on carriers, which are characterized by a durable and well-developed surface.
- Thermal combustion. Combustion is performed at an appropriately high temperature, which is maintained intentionally (e.g., by adding natural gas), usually in cases in which the use of a catalyst is not viable or impossible and if the composition of the gas being treated is variable. It is highly energy- and time-consuming.

Combustion processes ensure a relatively high percentage of removal of all kinds of organic compounds, to which the majority of odorants belong. The combustion process destroys the structure of the odor-generating compound, thus destroying its aromatic properties. Deodorization of gases using thermal oxidation may be used practically for all types of gases. Products generated during the combustion process can be neutralized during further stages of gas treatment. If the process is conducted correctly, it is very effective, makes it possible to obtain organic carbon concentrations below 20 mg/m^3^, which is difficult to achieve using biological methods, for example (Schlegelmilch et al. 2005b). The full combustion process can be generally described using the following dependencies (Eqs. 3 and Eq. 4):

3 $$ {\mathrm{C}}_{\mathrm{n}}{\mathrm{H}}_{\mathrm{m}}+\left(n+\frac{m}{4}\right){\mathrm{O}}_2\longrightarrow \mathrm{nC}{\mathrm{O}}_2+\frac{m}{2}{\mathrm{H}}_2\mathrm{O}+\mathrm{energy} $$

If other atoms are present in a molecule of combusted compounds (e.g., of sulfur, chlorine, fluoride, or nitrogen), also secondary pollutants occur. The basic disadvantages of thermal oxidation processes are economic considerations and the occurrence of secondary air pollutants (Kośmider et al. 2002; Schlegelmilch et al. 2005b). Basic secondary pollutants occurring during the combustion processes of hydrocarbons containing heteroatoms of sulfur, nitrogen, chlorine, and fluoride include (Kośmider et al. 2002; Schlegelmilch et al. 2005b):

- Sulfur oxides, thiols, sulphides, thiophenes, and sulfur
- Nitrogen oxides, pyridine, trimethylamine, acetonitrile
- Hydrogen chloride, trichloroethylene, dichloromethane, 1,1,1-trichloroethane, and dioxins
- Hydrogen fluoride, fluoroacetophenone, fluorophenol

**Fig. 5:**
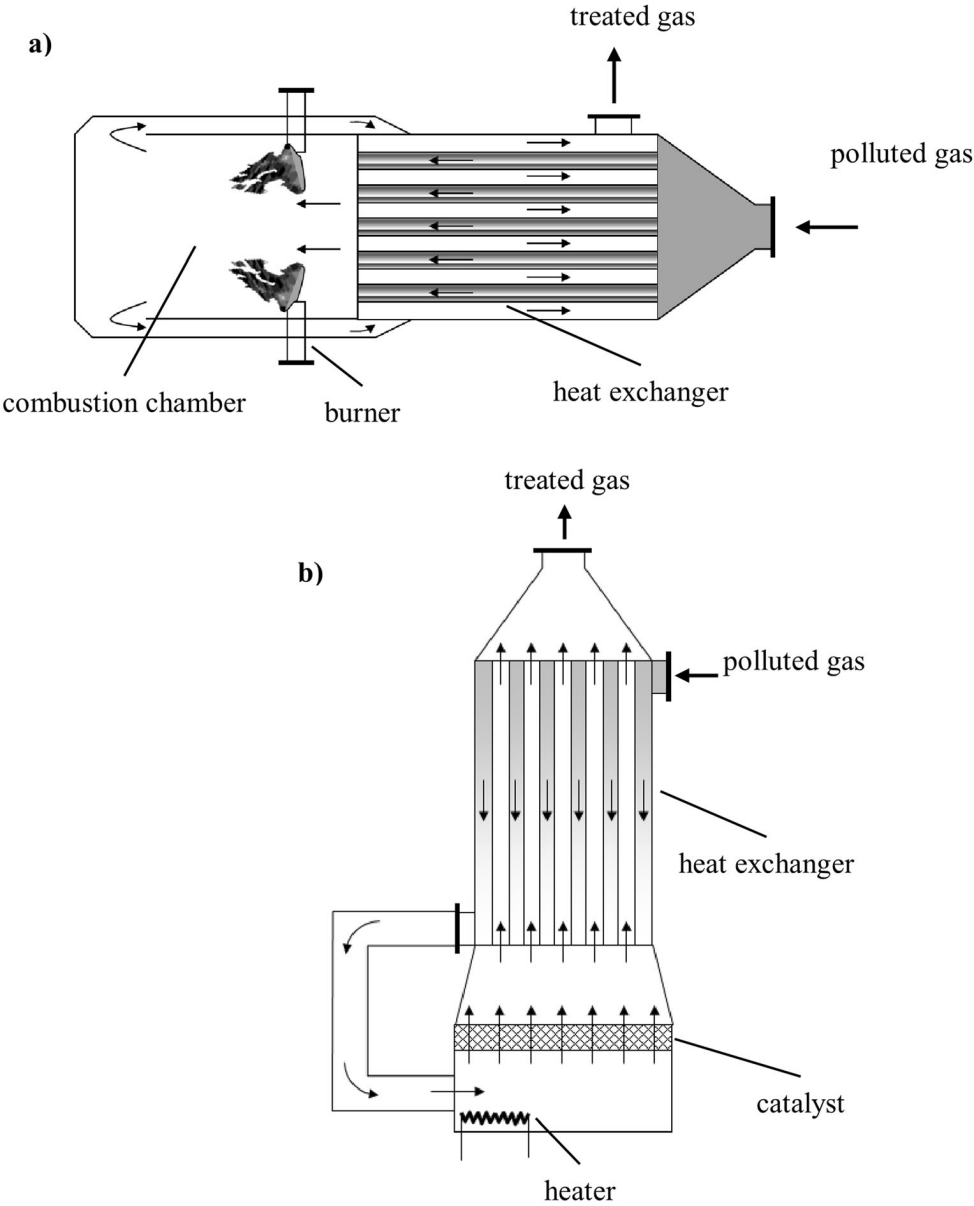
Diagram of the design of devices for thermal (**a**) and catalytic (**b**) combustion

Depending on the composition of the treated gas, there is the risk of corrosion and deposit formation on the surface of installation walls (Schlegelmilch et al. 2005b). Moreover, the content of organic compounds in gases subjected to deodorization usually does not exceed 1 g/m^3^, which makes it necessary to provide large quantities of energy. Figure 5 schematically presents design of selected devices for thermal neutralization of odor-generating substances.

Processes of catalytic oxidation of odorants gain increasing popularity due to savings in energy costs (Tsou et al. 2003). With the use of appropriate catalysts, the efficiency of the pollutant oxidation process in gases may reach almost 100% (Kwaśny and Balcerzak 2014). The use of catalysts reduces the activation energy of the combustion process (alternative substrate oxidation routes, the activation energy of which is lower) (Kolar and Kastner 2010) and increases the rate of reaction of compound decomposition (Kolar and Kastner 2010; Shie et al. 2005). The reduction in the reaction temperature reduces energy costs of the process and also reduces the production of greenhouse gases. Active substances that are usually used in catalysts include (Centi 2001; Kolar and Kastner 2010; Kośmider et al. 2002; Kwaśny and Balcerzak 2014; Tsou et al. 2003):

- Precious metals (e.g., platinum, palladium, ruthenium, iridium, and rhodium)
- Copper, cobalt, manganese, chromium, vanadium, wolfram, zinc, nickel, and iron oxides
- Oxides of elements of the LaMO_3_ type (a mixture of lanthanides combined with a mixture of nickel, cobalt or manganese)

Processes conducted with the use of catalysts can be successfully used if it is necessary to reduce emissions of ammonia, amines, hydrogen sulphide, sulphides, methanediol, dimethyl disulphide, benzene, chlorobenzene, toluene, o-xylene, or naphthalene (Kwaśny and Balcerzak 2014). Unfortunately, the cost of catalysts is also quite high (Kolar and Kastner 2010; Schlegelmilch et al. 2005b). To reduce costs, attempts are made to use also catalysts obtained from solid waste, e.g., fly ash from wood incineration processes (Klose et al. 2000; Kolar and Kastner 2010). Another method is the use of advanced combustion processes with energy recover, e.g., by means of recuperation (Schlegelmilch et al. 2005b). Thermal deodorization can also be combined with energy acquisition processes from burning of fossil fuels (Schlegelmilch et al. 2005b). A method that allows reduction of the quantity of energy needed for the combustion process is also enrichment of gaseous ingredients (e.g., adsorption on active carbon and next desorption). Gas deodorization with the application of combustion processes requires:

- An appropriate calorific value of gases (an appropriate quantity of combustible compounds)
- An appropriate amount of oxygen
- Obtaining an appropriate flash point or the use of a catalyst
- An appropriate residence time of a given ingredient in the reaction zone

Therefore, it is also necessary to provide an appropriate quantity of oxygen. Apart from oxygen from the air, oxygen itself can be also used as an oxidizing agent (Kolar and Kastner 2010). An appropriate time of residence of malodorous ingredients in the reaction zone is ensured if appropriate design solutions are used for the entire installation for thermal combustion, although the appropriate design of burners is the most important (Kośmider et al. 2002). However, from the point of view of green chemistry, environmental protection, and energy saving, the thermal processes seem to be of low attractiveness for the potential users (Szynkowska et al. 2009).

### Non-thermal gas oxidation

Non-thermal oxidation processes have also found application in the elimination of malodorous compounds. Oxidation processes are conducted using strong oxidizing agents (advanced oxidation processes—AOP), including hydrogen superoxide, ozone, hypochlorites, or chlorine (Biard et al. 2011; Charron et al. 2004; Couvert et al. 2006; Vega et al. 2014). Oxidation processes are also conducted in combination with absorption of malodorous gases in liquids. At that time, processes of malodorous substance oxidation occur in the absorption liquid. In this case, there still exists a broader range of oxidizing agents, e.g., using Fenton’s reaction (Vega et al. 2014). The group of methods based on non-thermal oxidation also includes processes based on UV radiation or plasma (Mielcarek et al. 2009; Yao and Feilberg 2015; Yet-Pole 2004; Zhu et al. 2017). The distribution of odor-generating substances may occur by direct oxidation of a stream of treated gases or by oxidation of substances absorbed in the sorbent in the scrubbers (Vega et al. 2014). Scrubbers are used, e.g., during treatment of malodorous gases containing organic sulfur compounds (Couvert et al. 2006; Vega et al. 2014). The odorant absorption process is conducted simultaneously with the process of generating highly reactive oxidizing agents, such as hydroxyl radicals for example (Couvert et al. 2006; Vega et al. 2014). Hydroxyl radicals are characterized by a higher oxidizing potential (2.8 eV) than, for example, hydrogen superoxide or chlorine (1.78 eV and 1.36 eV, respectively) (Lin and Chang 2005; Myslinski et al. 2000; Vega et al. 2014). The use of UV radiation (photooxidation) is a method of generating radicals or ions for oxidizing malodorous compounds contained in treated gases. However, it requires application of the UV generators consuming significant amount of energy, which substantially increases operation costs. To obtain considerable effects in gas treatment, it is required to use large amounts of energy and thus, considerable operating expenditures are necessary. For this reason, methodological solutions based on the use of UV radiation are not used willingly (Schlegelmilch et al. 2005b). Photooxidation, among other things, has found application also in deodorization processes of potable water (Zoschke et al. 2012). The combined use of UV radiation and an advanced oxidation process using hydrogen superoxide is used, for example, to remove malodorous compounds from gases, which are formed during wort production at breweries (Jurgens et al. 2007). This technological solution involves generation of hydroxyl radicals using UV radiation. Absorption of photon with a wavelength of 254 nm (UV) results in decomposition of the hydrogen superoxide molecule into two very reactive hydroxyl radicals (Vega et al. 2014) (Eq. 5):

5 $$ {\mathrm{H}}_2{\mathrm{O}}_2+ h\nu \longrightarrow 2{}{}^{\bullet}\mathrm{OH} $$

Pollutants are degraded both directly by hydrogen superoxide and by generated radicals (Vega et al. 2014). Depending on the nature of the pollution, degradation of a compound (odorant) occurs even above 99% of the content in treated gas (Jurgens et al. 2007). However, not all compounds are susceptible to such decomposition. The removal of dimethyl sulphide is quite challenging, for example (Jurgens et al. 2007). When Fenton’s reaction is used (Eqs. 6 and 7), during the oxidation of malodorous compounds absorbed in the absorption liquid, hydroxyl radicals are formed as a result of iron reaction with hydrogen superoxide (Pignatello et al. 2006, 2007; Vega et al. 2014):

6 $$ {\mathrm{Fe}}^{2+}+{\mathrm{H}}_2{\mathrm{O}}_2\longrightarrow {\mathrm{Fe}}^{3+}+{}{}^{\bullet}\mathrm{OH}+{\mathrm{O}\mathrm{H}}^{-} $$

7 $$ {\mathrm{Fe}}^{3+}+{\mathrm{H}}_2{\mathrm{O}}_2\longrightarrow {\mathrm{Fe}}^{2+}+{\mathrm{H}}^{+}+{\mathrm{H}\mathrm{OO}}^{\bullet } $$

A certain innovation of Fenton’s reaction is photo-Fenton’s reaction (Eq. 8), where radiation supports the catalyst regeneration process (Pignatello et al. 2006, 2007; Vega et al. 2014):

8 $$ {\mathrm{Fe}}^{3+}+{\mathrm{H}}_2\mathrm{O}+\mathrm{h}\upnu \longrightarrow {\mathrm{Fe}}^{2+}+{\mathrm{H}}^{+}+{}{}^{\bullet}\mathrm{OH} $$

**Fig. 6:**
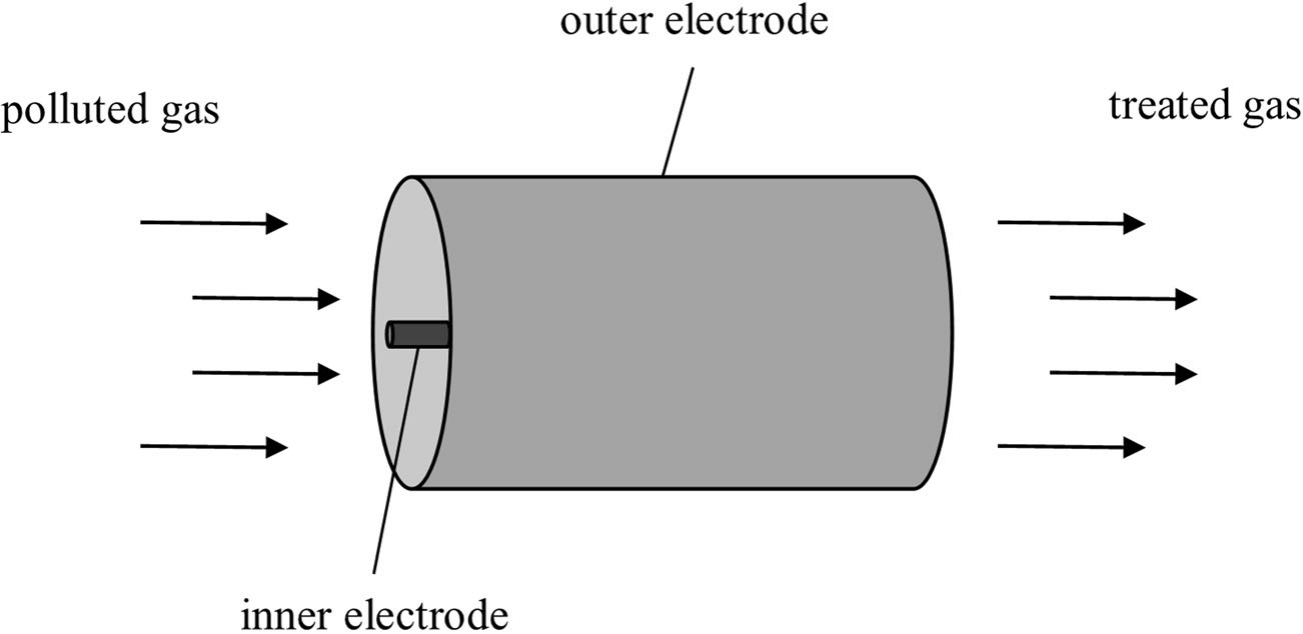
Diagram of the design of a reactor for generation of low-temperature plasma

A separate group of technological solutions includes photocatalytic processes: photocatalysts require activation radiation with a specific wavelength from the UV range (Yao and Feilberg 2015; Zhu et al. 2017). After activation, they are capable of low-temperature oxidation of malodorous compounds. Such catalysts include for example titanium oxide (TiO_2_), which requires activation with wavelengths from the 300–375 nm range, zinc oxide (ZnO), cerium oxide (CeO_2_), tungsten oxide (WO_3_), and cadmium sulphide (CdS). Dimethyl sulphide, phenols, chlorophenols, or alcohols are oxidized using the catalytic method (Kwaśny and Balcerzak 2014; Nishikawa and Takahara 2001). Many scientific units work on the methods of in-situ formation of hydroxyl radicals employing the electrodes (electrochemical oxidation), for example β-PBO_2_ electrodes are utilized for removal of phenol compounds and ethanethiol (Cong and Wu 2007; Ma et al. 2013). Also, low-temperature plasma (non-thermal, “cold”) has found application in deodorization processes (Fig. 6).

On the basis of literature data (Chang 2003; Jarrige and Vervisch 2007; Mizuno 2013; Oda 2003), it can be concluded that it is a technological solution, which certainly will become more popular. Plasma is generated during electric discharges between electrodes. Two types of discharges are possible—incomplete discharges—which do not cause a short circuit between the electrodes of the reactor or complete ones—arcing. Discharges cause ejection of electrons from atoms of the plasma gas. When low-temperature plasma is generated, electric energy is used for high-energy emissions of electrons and UV radiation. The gas itself remains “cold,” on average its temperature increases by approx. 10°. These electrons are accelerated in the magnetic field and high kinetic energy is obtained, they collide with further gas atoms, which result in the formation of further electrons and ions (chain reaction). Components occurring in the gaseous state are transformed into plasma. During collisions, energy exchange, dissociation, atomization, ionization, and molecule excitation occur. Plasma contains electrons, neutral particles, and ionized and free radicals. As a result of contact between plasma and pollutants, (also malodorous ones), degradation of these compounds occurs in the gas (Andersen et al. 2013; Mizuno 2013; Schlegelmilch et al. 2005b). Plasma has found application, among other things, in deodorization of gases from production of animal feed, fish feed, and from tobacco factories, especially if pollutants occur in very small amounts (Andersen et al. 2013; Vandenbroucke et al. 2011). When malodorous substances are present at high concentration level in flue gas stream, it is necessary to increase power of the devices, thus yielding significant increase in energy consumption. It is recommended that this technology should be used when concentrations of volatile organic compounds do not exceed 100 mg /m^3^ (Schlegelmilch et al. 2005b). The basic advantage of this technology includes a small size of devices and a small decrease in the pressure of the flowing gas (Andersen et al. 2013). Ozone is created when UV radiation is used. Care should be taken to neutralize it before releasing gas into the environment (Andersen et al. 2013; Schlegelmilch et al. 2005b).

### Biological gas treatment

If treated gases contain biodegradable components and do not contain substances, which are toxic to microorganisms used in the process, the deodorization process can be conducted using biological methods. Gases subjected to biological treatment must be characterized by the parameters, which guarantee biological activity of microorganisms (e.g., temperature, pH value) (Chen et al. 2016). If proper measures are applied to avoid excessively high pollutant concentration in the bioreactor, also substances toxic to the microorganisms can be handled using biological treatment methods (Barbusinski et al. 2017). Pollutants, which are to be removed by means of biological treatment must be soluble in the sorbent (which is an environment for the life of microorganisms), at least to a minimum degree. Water is usually the sorbent but pollutants can be also removed if they dissolve, for example, in fats, i.e., directly in lipids, which constitute the composition of the bacterial cell membrane. Selected pollutants removed with biological methods are presented in Table 2.

**Table 2:** Methods of biological deodorization of selected odor-generating pollutants

Pollutant	Deodorization method	Reference
Ammonia	Biofilters, biotrickling, bioscrubbers	Hansen and Rindel 1992; Hvidtfeldt Rasmussen et al. 1994; Weckhuysen et al. 1994; Joshi et al. 2000; Liang et al. 2000; Malhautier et al. 2003; Hong and Park 2004; Pagans et al. 2005; Phillips 2007; Yu et al. 2007; Sakuma et al. 2008; Nisola et al. 2009; Jiang and Tay 2010; Moussavi et al. 2011; Lee et al. 2013; Martel et al. 2013; Yang et al. 2014; Kawase et al. 2014; Kafle et al. 2015
Acetone	Biofilters	Lee et al. 2013
Dichloromethane	Biofilters, biotrickling	Diks and Ottengraf 1991; Yu et al. 2006; Phillips 2007; Jianming et al. 2014
Ethyl acetate	Biofilters, biotrickling	Hornos 2008; Sempere et al. 2009; Zare et al. 2012; Estrada et al. 2013
Hydrogen sulfide	Biofilters, biotrickling, bioscrubbers	Kanagawa and Mikami 1989; Zhang et al. 1991; Shinabe et al. 1995; Dijkman 1995; Kraakman et al. 1997; Nishimura and Yoda 1997; Janssen et al. 2000; Hansen and Rindel 2000; Hartikainen et al. 2001; Cox and Deshusses 2001; Elias et al. 2002; Deshusse and Cox 2003; van Durme et al. 2002; Morgan-Sagastume et al. 2003; Kim and Deshusses 2004; Gabriel and Deshusses 2003; Oyarzun et al. 2003; Malhautier et al. 2003; Deshusses and Gabriel 2005; Potivichayanon et al. 2006; Phillips 2007; Datta and Allen 2005; Yu et al. 2007; Aroca et al. 2007; Rattanapan et al. 2009; Ramírez et al. 2009; Jie et al. 2010; Jiang and Tay 2010; Park et al. 2011; Chaiprapat et al. 2011; Omri et al. 2013; Montebello et al. 2012; Wongwutthi and Limpaseni 2012; Liang and Liang 2013; Liu et al. 2013; Alfonsín et al. 2015; Ahmed et al. 2014; Ben Jaber et al. 2014; Chouari et al. 2015; Kafle et al. 2015; Santos et al. 2015; Kasperczyk and Urbaniec 2015; Hernández et al. 2016
Dimethyl sulfide	Biofilters	Kanagawa and Mikami 1989; Zhang et al. 1991; Smet et al. 1996; Smet et al. 1999; Shu and Chen 2008; Chung et al. 2010; Ben Jaber et al. 2014
n-Butanol	Biofilters, biotrickling	Lee et al. 2013; Szulczynski et al. 2018; Schmidt and Anderson 2017
Dimethyl disulfide	Biofilters	Kanagawa and Mikami 1989; Zhang et al. 1991; Cho et al. 1991; Ben Jaber et al. 2014; Bajpai 2014
Methanethiol	Biofilters, biotrickling	Kanagawa and Mikami 1989; Lee and Shoda 1989; Zhang et al. 1991; Montebello et al. 2012; Ben Jaber et al. 2014; Lebrero et al. 2014; Hernández et al. 2016
Ethanethiol	Biofilters	Kanagawa and Mikami 1989; Zhang et al. 1991; Ben Jaber et al. 2014
Diethanolamine	Biofilters	Moshrefzadeh and Sabour 2014
n-Hexane	Biofilters biotrickling	Zehraoui et al. 2013; Cheng et al. 2016
Phenol	Biofilters	Zilli et al. 1996
Styrene	Biofilters, biotrickling	Rene et al. 2009; Song et al. 2012; Runye et al. 2015; San-Valero et al. 2017; Gąszczak et al. 2018
beta-Pinene	Biofilters	Viswanathan et al. 2013
Limonene	Biofilters	Viswanathan et al. 2013
Tetrachloroethylene	Biofilters	Devinny et al. 2009
Toluene	Biofilters, biotrickling	Weber and Hartmans 1995; Hwang and Tang 1997; Delhoménie et al. 2001; García-Peña et al. 2001; Woertz et al. 2002; Cox & Deshusses 2000; Tresse et al. 2003; Sakuma et al. 2006; Phillips 2007; Bhaskaran et al. 2008; Hornos 2008; Yang et al. 2010; Dorado et al. 2012; Lebrero et al. 2012; Saucedo-Lucero et al. 2014
Trichloroethylene	Biofilters	Devinny et al. 2009
Xylene	Biofilters	Amin et al. 2014
Carbon disulfide	Biotrickling	Lobo et al. 2000
Chlorobenzene	Biotrickling	Yang et al. 2013
Ethylbenzene	Biotrickling	Wang et al. 2014
Formaldehyde	Biotrickling	Prado et al. 2006
Methyl tert-butyl ether	Biotrickling	Fortin and Deshusses 1999

Biological treatment of malodorous substances present in the gas stream under purification results from two processes: sorption of polluted gases and their biological decomposition. First, the sorption process occurs, as a result of which malodorous substances are removed from the gas. Next, the biological decomposition process of absorbed pollutants occurs—sorbent regeneration. Biological decomposition takes place as a result of the activity of microorganisms, which are selected depending on the type of ingredient to be removed from the gas stream (Hernandez et al. 2012). In this way, microorganisms gain energy and metabolites necessary for their life processes. Depending on the method of conducting the biological process of gas treatment, microorganisms can be suspended in the sorbent (bioscrubbers) or set on the surface of a solid material (biofilters). Bioreactors of different design can provide quantitative course of the gas purification process. Such methodological solutions are perfect for purification of the flue gases containing SO_2_ at the concentration level of 300–1000 ppm (Lin et al. 2015), as well as for removal of the odors originating from sewage treatment plants (Accortt et al. 2001; Alfonsín et al. 2015; Lucernoni et al. 2016; Zhou et al. 2016) or from breeding farms. Due to low costs, the biological processes belong to the methods, which are the most frequently utilized for removal of odor-generating pollutants, in spite of one of the highest operation hazards (Estrada et al. 2012; Lebrero et al. 2010).

Despite many similarities, there are substantial differences between biofilters and bioscrubbers. The advantages and disadvantages of both deodorization methods are presented below (Table 3).

**Table 3:** Advantages and disadvantages of biological methods of deodorization of gases

	Bioscrubbers	Biofiltration
Advantages	- Simple technology - Low operation costs - Small pressure drops of flowing gas - Possibility of acidic gases deodorization	- Simple technology - Low operation costs - Low investment costs - Possibility of implementation in thorough gas purification processes - Low-waste technology - Possibility of application of filtering materials with strong adsorption properties
Disadvantages	- Necessity of application of suitable nutrients for microorganisms - Necessity of reagents dosing, for example in order to correct pH - Necessity of excess biomass removal	- Replacement of filtering material every 2–5 years - Difficulties with deodorization of gases with high pollutants content - Difficulties with pH correction (frequent need for buffering substances) - Necessity of bed humidity control - Necessity of bed oxygenation in case of purification of gases lacking oxygen - Problems with bed overgrowing - High spatial demands of the installation

#### Bioscrubbers

Gas deodorization with the use of bioscrubbers involves transporting the mass of pollutants from the gaseous phase to the adsorption liquid, which contains suspended microorganisms (this is usually an aqueous suspension of activated sludge). This process is conducted mostly with countercurrent or if bioscrubbers are packed with a cross-flow. To increase the contact surface between the liquid and gaseous phases, appropriate packing is often used (Schlegelmilch et al. 2005b). The packing is covered with a biofilm layer with microorganisms. Degradation of pollutants occurs owing to microorganisms suspended in the absorption liquid or living in the biofilm. The number of microorganisms increases in the liquid phase in this suspended form as a result of degradation of impurities or the volume of the biofilm increase on the surface of packing. Excess microorganisms should be removed on a regular basis. If bioscrubbers contain packing, it cannot become overgrown. For this purpose, packing with large pores should be selected and the deposit should be rinsed frequently. Good results are obtained by using movable packing, e.g., suspended in the fluid phase where elements of packing collide with one another, thus removing excess biofilm from their surface. Bioscrubbers coupled with an activated sludge tank are another solution. The sorbent regeneration process occurs in the activated sludge tank. The use of an additional tank makes it possible to extend the duration of the biodegradation process, i.e., to extend the sorbent regeneration time. Sorbent regeneration can be intensified by extending the age of the sludge or using special treatments, e.g., aeration or the addition of deficient nutrients or substances regulating the pH value of the sorbent (Kośmider et al. 2002; Schlegelmilch et al. 2005b). The linear speed of the gas stream flow through bioscrubbers usually falls between 0.5 and 2.5 m/s. In packed bioscrubbers, sorbent irrigation in an amount of approx. 20–60 m^3^ per m^2^ of the packing over an hour is used on a standard basis (Schlegelmilch et al. 2005b). Similarly to the absorption methods,this approach also employs barbotage bioreactors where the gas stream under purification constitutes a dispersed phase and the liquid with microorganisms is a compact phase (Lebrero et al. 2010). This method can be used to purify the gas stream of relatively low flow rate.

#### Biofiltration of malodorous substances

**Fig. 7:**
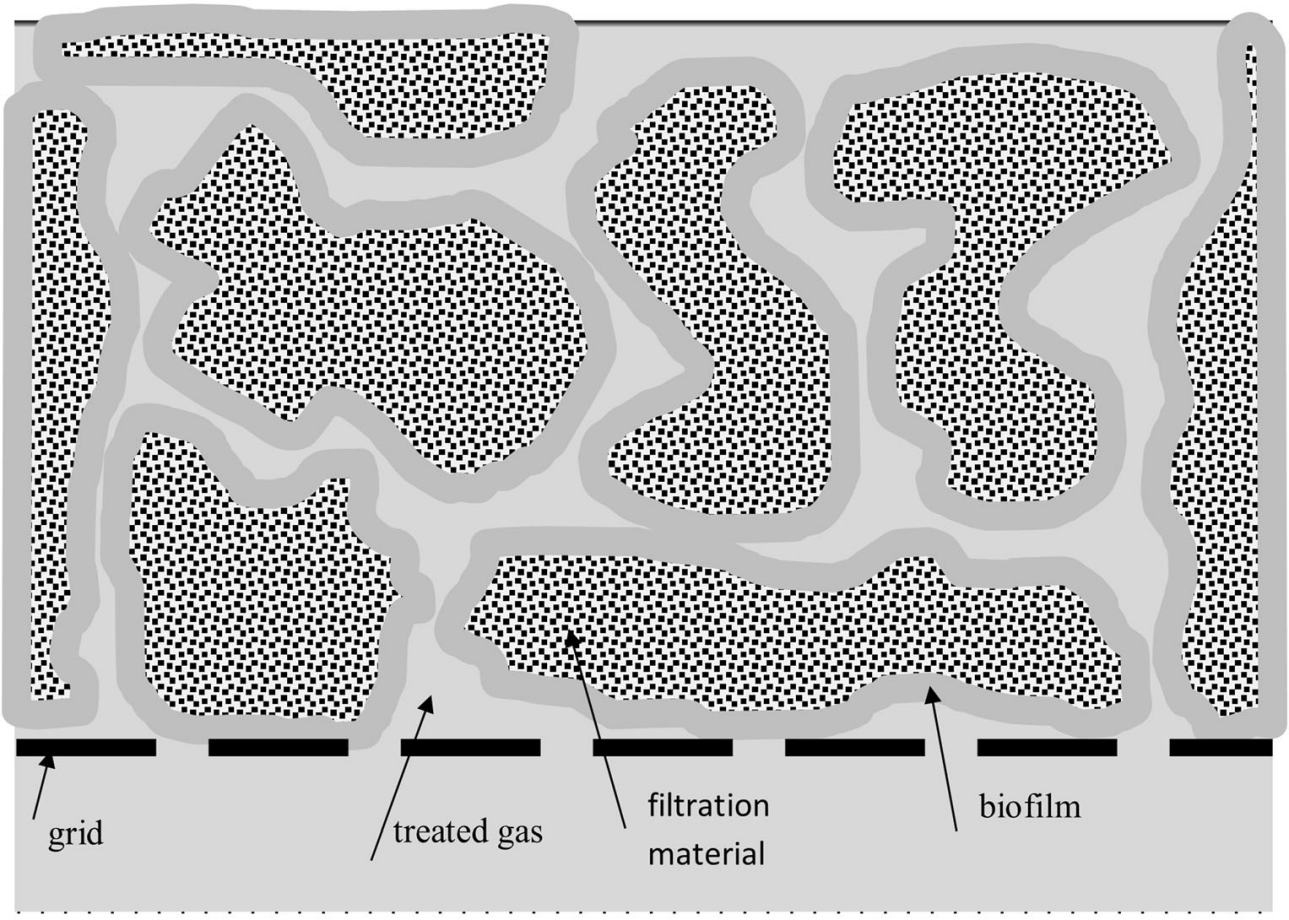
Biofilter packing

Another frequently utilized method based on the biological processes is biofiltration. It is a process of removing pollutants with the use of microorganisms living in the filter packing (solid deposit—porous material). Microorganisms, which live on the surface of the porous material, which constitutes the filling of the filter, form biological film (Lebrero et al. 2013) (Fig. 7).

Biological film absorbs pollutants from the gas flowing through the filter packing. Microorganisms, which are present in the biofilm, as a result of biological processes, contribute to their decomposition to odorless substances (preferably to CO_2_ and H_2_O) (Schlegelmilch et al. 2005b) or build them into their cell structure (Deshusses 1997). If biofilters are used in gas treatment processes (also malodorous ones), it is very important to ensure appropriate conditions for the development of microorganisms, which settle the packing (Easter et al. 2005; Lebrero et al. 2013). Moisture of the deposit is one of the most important parameters. Optimally, it should range from approx. 30 to 60%. Usually, a controlled deposit hydration process is conducted using an irrigation system. To reduce the drying effect, if possible, the gas, before treatment in biofilters, goes through scrubbers (possibly bioscrubbers) with an aqueous phase as an absorbent. This ensures appropriately high moisture of the stream of gas introduced into biofilters (Schlegelmilch et al. 2005b). Another method involves mixing of the stream of treated gases with a stream of water vapor. Excess water is collected in the form of condensate and is directed, e.g., to hydration of the filtration deposit (Wierzbińka and Modzelewski 2015). Stream of gases directed to biofiltration should be characterized by relative humidity of 95–100% (Wierzbińka and Modzelewski 2015). However, the packing moisture should not be too high. This contributes to too high resistance in the flow of the treated gas. Excessive moisture of the deposit may cause formation of anaerobic zones, thus interfering with microorganism development (Kennes and Thalasso 1998). Microorganisms living in the deposit require appropriate temperatures. Care should be taken to ensure that the temperature of treated gases does not cause fluctuations in the deposit temperatures. The optimal value of the majority of microorganisms falls between 30 and 40 °C (Kośmider et al. 2002; Schlegelmilch et al. 2005b). Proper growth of microorganisms also depends on pH of biological membrane (Ralebitso-Senior et al. 2012). Optimal pH value, depending on the type of microorganisms inhabiting the membrane, equals 5–7 (Schlegelmilch et al. 2005b), 7–8 (Wierzbińka and Modzelewski 2015). The optimal pH value depends on the kind of microorganisms, which can live in the deposit. Therefore, treatment of gases containing large amounts of organic and inorganic sulfur or nitrogen compounds that are acid precursors becomes a problem when this method is used (Schlegelmilch et al. 2005b). Despite this, it is possible to conduct biofiltration of such aggressive gases by introducing appropriate microorganisms into the filtration material (previous deposit preparation) (Ben Jaber et al. 2016). Further important aspects include availability of biogenic elements and proportions between them (C:N:P. = 100:5:1), absence of poisons (e.g., strong oxidizing agents, heavy metal ions, cyanides, or detergents), and limitation of harmful UV radiation (with a wavelength falling within the range of 230–275 nm) (Kośmider et al. 2002). It is also important to ensure appropriate pressure of the flowing gas so as to distribute it evenly over the entire cross-section of the biofilter (Schlegelmilch et al. 2005b). In case of slow changes of magnitude of the parameters, the microorganisms have a chance to adapt. They are capable of adapting to small changes. Therefore, the parameters of the gas stream should be maintained as well as dispensing of the liquid stream. The linear gas stream rate through the deposit usually falls within the range of 0.03–0.1 m/s (Kośmider et al. 2002). This is aimed at limiting the possibility of the occurrence of anaerobic zones and the formation of channels, through which the gas stream could by-pass the biological membrane. Such a flow ensures the treatment of 40 to 500 m^3^/(m^2^ h) of gas (Kośmider et al. 2002;; Schlegelmilch et al. 2005b). Biofiltration requires application of the deposit with developed surface area in order to enable development of the biological membrane (Gutarowska et al. 2014). Filtration deposit should guarantee a large surface of contact with a low decrease in the pressure of the stream of flowing gas. The deposit should be characterized by a large surface area, a proper, loose structure, and low resistance of the treated gas flow. The most convenient situation occurs when the deposit consists mainly (of 55% order) of organic material, when the packing diameter *d*_50_ is greater than 4 mm, and the volume of pores in the grains is higher than 90% (Wierzbińka and Modzelewski 2015). The rate of pollutant mass exchange between the treated gas and the biofilm, i.e., the gas treatment rate, will depend on the biofilm surface. Both organic materials, i.e., compost, peat, tree bark, woodchips, straw, and even soil (Schlegelmilch et al. 2005b), and inorganic ones, e.g., bentonite or perlite (Gutarowska et al. 2014), are used as packing. Filtration material should be durable enough to provide minimum condensation of the deposit (decrease in its specific surface area) with operation time (Wierzbińka and Modzelewski 2015). In this case, inorganic materials reveal superior parameters as far as the process of malodorous ingredients removal is concerned (Ben Jaber et al. 2016). The depth of the deposit is usually up to 2 m. The deposit must be suitably prepared, i.e., time must be devoted to settle appropriate microorganisms in it. Such a process can last a few days, even up to several weeks (Streese et al. 2005). This process can be accelerated by prior inoculation of the deposit (Kennes and Thalasso 1998; Schlegelmilch et al. 2005b). The biofiltration process can be conducted both in open and closed filters. The effectiveness of biofiltration conducted in open filters strictly depends on the climatic conditions. The geographical regions characterized by high ambient air temperature and low humidity suffer from a problem of excessive drying of deposits. This makes it necessary to irrigate biofilters. While in the regions with a large amount of precipitation, the problem of excessive moisture of filters occurs. In this case, it is necessary to protect them from precipitation (Accortt et al. 2001). Closed filters are less dependent on atmospheric changes and they ensure better distribution of moisture inside filters (even during wet season). The technologies of gas deodorization based on biofiltration processes can be successfully employed for purification of the gases susceptible to biological decomposition, e.g., landfill gases from wastewater treatment plants, process gases from food production that contain large amounts of organic components (Gutarowska et al. 2014; Huber-Humer et al. 2008; Schlegelmilch et al. 2005a). Effectiveness of the purification process depends on type of filtration material, type of ingredients present in gas stream to be purified and parameters of biofiltration process. Thus, in the case of H_2_S removal, it ranges from 50% when sapwood (a part of tree trunk conducting water with mineral salts) is used as a biofiltration material, even up to 100% when compost or peat with selected microorganisms is the filtration material (Dumont and Andres 2010; Ma et al. 2006; Oyarzun et al. 2003). The biofiltration of gases from the wastewater treatment plant is conducted on compost, which makes it possible to achieve efficiency of over 90% for the compounds such as limonene, ketones, or benzene, above 80% for toluene and dimethyl sulphide (Lebrero et al. 2013). Elements of a biofilter bed are moisturized with water, so the biofilm formed on the surface of these elements allows for the absorption of hydrophilic compounds. The effectiveness of the removal of these types of compounds in biofilters depends on the rate of their degradation by microorganisms present in the biofilm. The situation is different in the case of compounds having greater affinity to the organic phase than to the aqueous phase. Biofiltration of hydrophobic compounds proceeds at a much lower yield than hydrophilic compounds, and the effectiveness of biofiltration depends on the rate of mass transport of the hydrophobic component from the gas phase to the biofilm. The improvement of biofiltration of hydrophobic compounds is a challenge in biofilter design and it is currently the main research topic in this area (Cheng et al. 2016; Ferdowsi et al. 2017). In addition, attention is paid to the interaction between the groups of compounds for synergistic or inhibitory effects of their simultaneous removal from gaseous streams.

Summing up, the aforementioned deodorization techniques are presented in Table 4 comparing mean efficiency of removal of fundamental malodorous pollutants using different deodorization techniques (Webb 2016).

**Table 4:** Mean efficiency of removal of malodorous pollutants using different deodorization techniques

Methods of gas deodorization	Hydrogen sulphide	Ammonia	Reduced sulphides	Odor	VOC
	Performance				
Chemical absorption	99%	99%	20–50%	50–75%	Negligible
Adsorption (activated carbon)	98–99%	60–70%	50–85%	> 90%	> 95%
Thermal oxidation	98%	98%	98%	> 95%	98%
Biotrickling filters	> 99%	Negligible	20%	75–90%	Negligible if water insoluble
Biofilters	> 99%	90%	75–98%	90%	Up to 95%

**Fig. 8:**
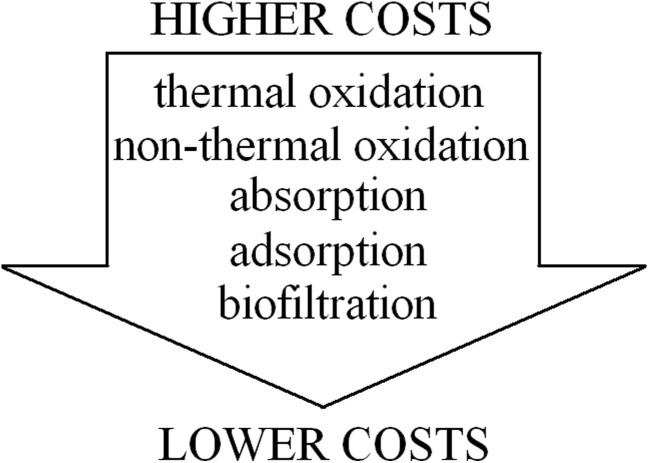
Schematic diagram presenting operating cost of particular deodorization technique

A very important factor deciding about the way odorous pollutants are removed from given installation is the total cost of particular process (Barbusinski et al. 2017; Bindra et al. 2015; Estrada et al. 2011). Figure 8 schematically illustrates a sequence of deodorization techniques depending on their operating cost.

A comparison of described deodorization techniques with respect to initial costs, operating costs, and maintenance costs of particular installation is shown in Table 5.

**Table 5:** Comparison of exploitation costs for particular deodorization techniques

Criteria	Absorption	Adsorption	Thermal oxidation	Non-thermal oxidation	Biofiltration
Initial cost	Low	Low	Moderate	Moderate	High
Operating cost	High	High	High	High	Low
Maintenance cost	High	High	Moderate	Moderate	Low

### Introduction of admixtures changing the character of the odor

**Fig. 9:**
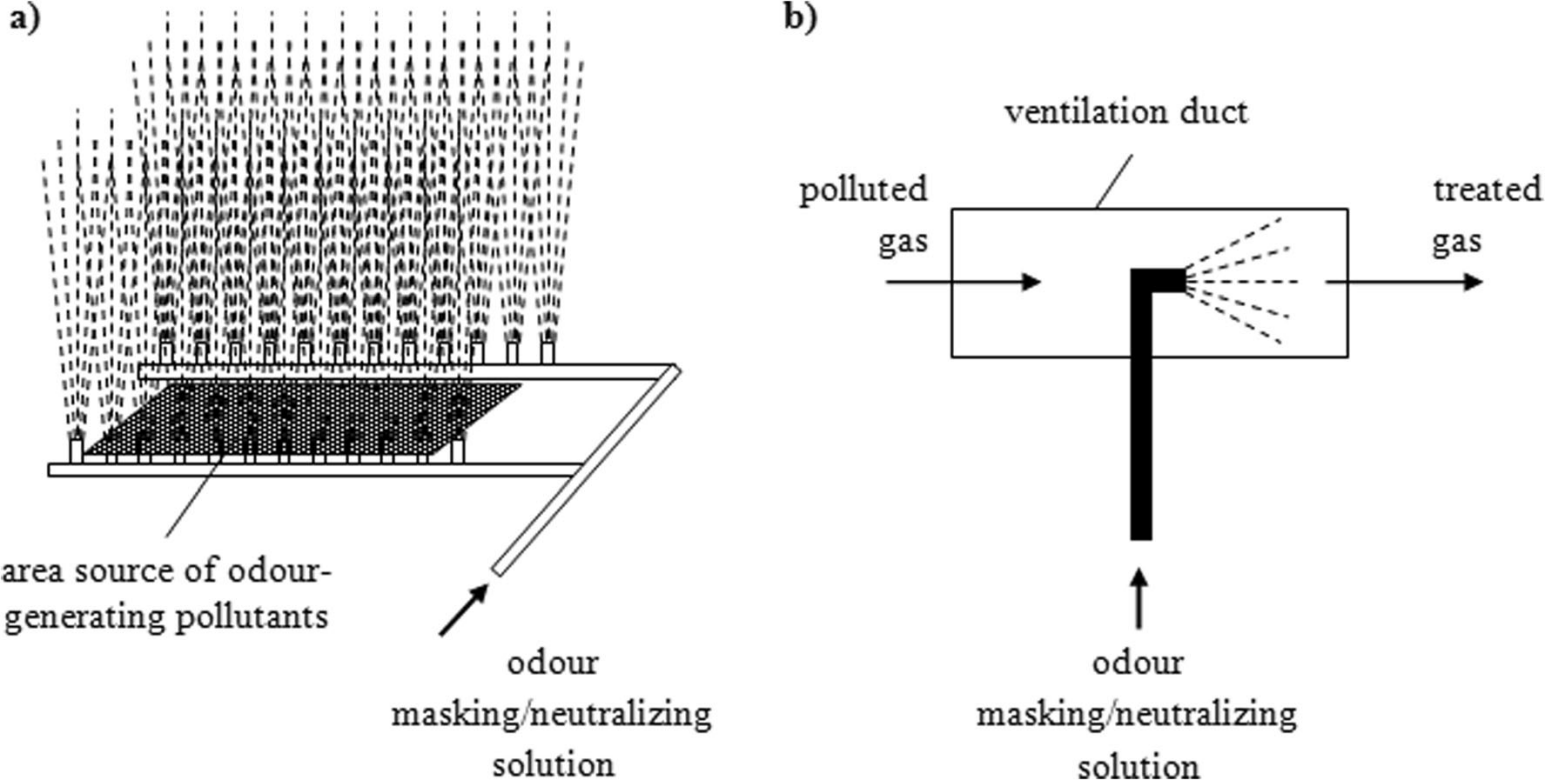
Devices used for deodorization of gases by introduction of admixtures changing the character of the odor: **a** antiosmic barrier; **b** spraying masking liquid in the ventilation duct

While performing measurements of the concentration of individual ingredients in the odorant mixture, it is difficult to predict odor intensity. This is connected with the occurrence of phenomena such as synergism, neutralization, and odor masking. These are olfactory interactions between gas ingredients. The intensity of the odor of a mixture is usually completely different from that which was predicted on the basis of olfactory thresholds of particular ingredients present in the gas stream and the values of their concentration in this gas. If it is higher than predicted, it is the so-called odor synergism (enhancement of an odor as a result of mutual interactions of two or a larger number of ingredients on each other). If odor intensity is lower than predicted, it is so-called neutralization (compensation) of an odor (reduction in odor intensity as a result of mutual interactions of two or a larger number of ingredients on each other). Masking, on the other hand, is replacement of an unpleasant odor with a pleasant one, i.e., introduction of an ingredient, which changes the nature of the odor into the mixture. The introduced ingredient blocks unpleasant odors leaving only a pleasant stimulus (Kośmider et al. 2002). Research on odor compensation was undertaken already over 100 years ago (Wise et al. 2000). These phenomena are used during introduction of admixtures to the stream of malodorous gases. Such a solution has found application in some cases when odorants occur at low concentrations and when they are not toxic. The process involves introduction into the stream of gas (or into a room in which odors are pleasant) the admixtures, which change the nature of the odor (Fig. 9) or its intensity (Mielcarek et al. 2009; Piecuch et al. 2011).

Various air fresheners, which are installed at public utility facilities or in ventilation and air-conditioning systems, use this principle. The so-called walls of mist (antiosmic barriers (Fig. 9)), which cover facilities emitting onerous odors, may also contain such compounds (Kośmider et al. 2002). They have found application in wastewater treatment plants, landfills, and food processing plants (Andriyevska et al. 2008). Masking or odor neutralization processes are also a good alternative for supplementary treatment of exhaust gases. In the odorant mixture, the removal of the majority (90%) of pollutants does not always guarantee weakening or elimination of an unpleasant odor. It is possible that opposite effects will appear, i.e., we will observe an increase in the intensity of the odor despite reduced concentrations of individual odorants in the mixture. This can be caused by, for example, transformation of a certain group of odor-generating compounds into substances with a lower olfactory threshold. Such a phenomenon occurs, for example, during oxidation of alcohol-containing gases (olfactory thresholds of alcohols are on average 1000 times higher than those for carboxylic acids formed in the oxidation process) (Kośmider et al. 2002). If a small amount of odor-generating pollutants remains in gases, which are not toxic, their odor can be neutralized or masked by the addition of appropriate admixtures. However, it is not the approach, which solves the problem of environmental protection (Lewkowska et al. 2016). Despite social discontent decline, the pollutants will be still present in the ambient air. As compounds, which are used as admixtures changing the character of the odor, mostly essential oils are used, e.g., eucalyptus oil, lemon oil, or Siberian fir tree oil. Unfortunately, their effectiveness is sufficient only in case of the ingredients characterized by small values of odor concentration. Essential oils cannot block stimuli induced by high concentrations of odorants (Kowalczyk and Piecuch 2016; Kowalczyk et al. 2013; Piecuch et al. 2015). The introduction of an appropriately large amount of the masking substance might result in too intensive odor of this substance and subsequent discomfort for recipients. Additional controversies arise from the fact that these solutions are not verified with respect to chemical and health safety. Admixtures can mask so-called warning odors; they may cause allergies and contaminate the skin and other surfaces. The use of such a method requires accurate verification due to the complexity of chemical reactions, which may accompany the introduction of a given agent into the mixture of treated gases. Decision about application of appropriate odor compensating agent must be preceded by experience in effectiveness of its action.

## Recommendations and future research

Due to a lack of corresponding legal regulations or unregulated legal status concerning odor nuisance in many countries, a fundamental step is objective evaluation of unpleasant odors impact on the environment and on life quality of people. The influence of unpleasant odor on odor nuisance can result from a couple of its characteristic features described by the FIDOL acronym (Loriato et al. 2012):

- Frequency of the odor occurrence
- Intensity
- Duration of the exposure
- Offensiveness of the odor—subjective
- Location of the odor

One of the most important problems connected with odor is duration of human exposure to unpleasant odors, which does not cause odor nuisance. Two concepts of solution of this problem are often accepted:

- Permanent or periodical limitation of emission level of the compounds characterized by unpleasant odor
- Short-lasting release of higher concentration of the compounds with unpleasant odor to minimize duration of the exposure.
- In order to prevent deterioration of ambient air quality, the countries which introduced legal regulations concerning air quality with respect to odor nuisance defined the emission standards implementing:
- Odor unit (ou/m^3^) allowing determination of concentration of particular odorants or their mixture (*c*_od_)
- Standards pertaining to odor emission (*q*_od_ [ou/h])
- Diagrams of the minimum distance between emission sources and residential areas
- Analysis of the complaints about odors

The European Union documents and different legal regulations concerning odor nuisance recommend application of the best available technologies (BAT) in production practice.

The Best Available Techniques (BAT) Reference Document (BREF) for Common Waste Water and Waste Gas Treatment/Management Systems in the Chemical Sector, which constitutes an update mandated by the Directive 2010/75/EU, outlines the procedures for minimizing the emission of malodors to the atmosphere (Brinkmann et al. 2016). These procedures include, among others, reduction of the waste storage time in aerobic conditions, the use of chemical reagents to reduce the formation of odorous compounds, and implementation of end-of-pipe treatment. Thermal oxidation, adsorption, and biofiltration were shown to be the most effective end-of-pipe techniques for reducing the emission of odors with up to 99.9% purification. It is suggested in the document that there is a possibility to combine the use of biofiltration and bioscrubbing as complementary techniques. This would entail the addition of few chemical agents and low-energy consumption. It should be noted however that selecting treatment techniques depends on many factors such as the concentration of odorants and volume of emitted gas, energy consumption, initial and operational costs of the installation, and available space. For instance, the use of an alkaline oxidative scrubber enables reaching high efficiency when aromatic odorants or amines are concerned, but as a result of the use of large volumes of strong oxidants is relatively expensive, apart from the cost of the reagents, there is a need to use special construction materials and waste treatment. Additionally, the authors of the document suggest sealing the hydraulic and pneumatic circuits of the installation. Due to extremely low odor thresholds of certain substances, such as sulfur compounds, dedicated solutions should be used instead of standard pipe fittings and centrifugal pumps. It seems that last decades witnessed significant increase in interest in application of biofilters for purification of the gas streams containing odorous compounds. Undisputable advantages of the biofilters are relatively low cost of their operation, low energy consumption, and relatively small number of the by-products formed (Abraham et al. 2015). It is very important from the point of view of environmental protection. A significant attention is attracted by general progress in biological techniques, which are promising as far as an increase in effectiveness of odorous compounds removal from gas mixtures is concerned. An interesting solution seems to be application of the biofilters’ packing of natural origin (a mixture of coconut fiber and sludge based carbon, peat and heather, and pine bark) (Alfonsin et al. 2013). Such solutions are an interesting approach to removal of malodorous compounds from gas streams due to their low negative impact on the environment.

## Conclusions

As a result of growing social awareness, dealing with the problem of odor nuisance related to manufacturing, service, breeding activities often becomes obligatory. The best solution is preventing emissions of odor-generating substances. Unfortunately, this is not often possible. Therefore, effective deodorization technologies for malodorous gases are being looked for. There are different solutions available, which are based on the following processes: absorption, adsorption, thermal neutralization, non-thermal oxidation, biological processes, or introduction of the admixtures that change odor character. Obviously, there is no ideal solution. Each technology possesses some advantages as well as certain drawbacks and limitations. Table 6 presents the advantages and disadvantages of the aforementioned deodorization techniques intended for the compounds characterized by odor nuisance.

**Table 6:** Advantages and disadvantages of the most frequently used gas deodorization technologies

Deodorization technologies	Disadvantages	Advantages
Absorption	- Problem of absorbent regeneration or disposal remains - Corrosion of installation - Possible secondary emissions whose source is used absorbent - High costs of pumping - If additional chemicals are used, they must be topped up in a controlled manner - Further stages of gas treatment are often required	- Low investment costs - Low operating costs - Treatment of gases containing high odorant concentrations - Possibility of recovery of adsorbed compounds
Adsorption	- Problem of adsorbent regeneration or disposal - Necessary to regenerate a deposit using large amounts of gases (subsequent dilution of pollutants) - Possible secondary emissions whose source is used adsorbent - It is often only one of gas treatment stages	- Possibility of recovery of adsorbed compounds
Thermal neutralization	- A high content of inflammable pollutants is required - Generation of secondary pollutants - High operating costs due to the necessity of gas enrichment or catalyst addition - Risk of corrosion and deposits on the installation - High energy consumption of the process	- Ensures high effectiveness of deodorization - Possibility of deodorizing gases with a broad range of odor-generating compounds - Waste-free process - Simple design and operation of the installation
Non-thermal oxidation	- Risk of corrosion of the installation - Operation with strongly oxidizing agents - It is often necessary to remove ozone from treated gases (e.g. after ozonisation processes or UV processes) - Processes are often highly energy-consuming (it is necessary to use UV generators using high amounts of energy) - It is possible to treat only compounds which are susceptible to oxidation (it is difficult to remove dimethyl sulfite)	- Low operating costs - Low investment costs - Waste-free process - Disinfection of treated gases is often an additional process - The small size of devices and a small decrease in the pressure of the flowing gas (plasma technology)
Biological gas treatment	- Treated gases must contain biodegradable components - Treated gases must not contain toxic substances - Treated gases must be characterized by parameters guaranteeing biological activity (pH, temperature, presence of acid precursors) etc. - Problem with an excessive amount of biomass—installation overgrowth - For biofiltration—a large surface area of the installation - Stability of gas treatment parameters	- Low operating costs - Low investment costs - High effectiveness when biological material is well selected - Possible to treat gases with low odorant concentrations
Introduction of admixtures changing the character of the odor	- Can be used only for non-toxic odorants - Treated gases must contain only low odorant concentrations - Possibility of weakening defense reactions of people exposed to the substances - High dependence on weather conditions (temperature, force and direction of wind)	- Low investment costs - Easy operation - Immediate effect

In absorption and adsorption processes, the problem of malodorous pollutants is transferred from one center (treated gas) to another one (sorbent—liquid or solid). Therefore, it necessary to couple these processes with appropriate sorbent regeneration (or possibly disintegration) processes. Absorption makes it possible to treat gases with much higher odorant concentrations than adsorption processes. However, if very low pollutant concentrations in the treated gas are required, further stages of treatment must be used (additional treatment). In the case of combustion processes, even 100% of deodorization effectiveness can be obtained. However, these processes are very expensive (due to energy consumption or the necessity to use catalysts). Low-temperature oxidation processes are not cheap either. An additional problem may be secondary products, which pollute the environment, e.g., ozone. The use of biological methods based on bioscrubbers and biofilters is developing dynamically. These are the methods, which actually combine sorption processes with biological processes. Generally speaking, they are characterized by relatively low operation cost. However, their application in technological practice requires construction of large-size devices, which is not always feasible to implement. Separate groups are the methods based on admixtures changing the character of the odor. However, their application is limited to the case when the odor concentration is not too high and their application causes a lot of controversy due to the possibility of masking substances, which are harmful to health.

Selection of suitable deodorization technique depends on:

- Techniques implemented in the plants having similar production profile
- Intensity of odorous substances emission
- Character of emitted substances
- Total content of pollutants
- Presence of odor (or surrogates of odor) regulations for ambient air quality
- Presence of nuisance (community) odor regulations (i.e., citizens have legal options to register complains),
- Site-specific history of complaints, legal challenges, public relations with the surrounding community/neighbors
- Available budget and economic analyses
- Knowledge about a particular technology applicable to a specific source

Presented critical analysis of available deodorization techniques intended for odorous pollutants leads to the conclusion that in each case, it is necessary to verify satisfactory deodorization effectiveness of the technique selected for particular technological process. It requires determination of odor intensity and hedonic quality at the inlet and at the outlet of an installation. Described deodorization techniques slightly differ from the generally known and utilized techniques for removal of other air and exhaust gas pollutants. Important differences stem from specific destination of the installations for deodorization. Satisfactory attenuation of odor can be achieved without a decrease in concentration of quantitatively dominant pollutants; it is enough to remove the ones, which possess unpleasant odor. However, it is often the case that high effectiveness of removal of particular group of odorants does not provide deodorization of the gases polluted with multicomponent mixture. That is why investment decisions must be preceded by direct tests performed on experimental or pilot installations. Removal of 90% of pollutants does not have to mean elimination or attenuation of odor, even opposite effects are possible—an increase in odor intensity of mixture. Obvious reasons of such phenomena are conversions of some pollutants into the products characterized by lower olfactory threshold or removal of the constituents, which neutralize odor in the initial mixture. Summarizing, selection of the most effective deodorization method is a difficult task and depends on many factors, among which installation cost can be dominant.
